# Effects of acute and long-term exercise modalities and doses on brain-derived neurotrophic factor in patients with stroke: a Bayesian network meta-analysis

**DOI:** 10.3389/fnsys.2026.1905191

**Published:** 2026-07-24

**Authors:** Hao Yang, Yan Rao, Lijun Chen

**Affiliations:** 1 Department of Physical Education, Tongji University, Shanghai, China; 2 Shanghai Normal University Tianhua College, Shanghai, China; 3 School of Sports and Health, Shanghai Lixin University of Accounting and Finance, Shanghai, China

**Keywords:** brain-derived neurotrophic factor, dose–response relationship, exercise, neuroplasticity, stroke

## Abstract

**Objective:**

To systematically evaluate the effects of exercise interventions on brain-derived neurotrophic factor (BDNF) in patients with stroke, and to examine the dose–response relationships associated with acute and long-term exercise.

**Methods:**

PubMed, Embase, Web of Science, the Cochrane Library, and APA PsycINFO were searched for studies of exercise interventions in patients with stroke that reported BDNF outcomes. Risk of bias was assessed using RoB 2.0 and ROBINS-I. A network dose–response meta-regression was conducted within a Bayesian random-effects framework.

**Results:**

Twenty-three studies involving 903 participants were included, comprising 14 randomized controlled trials, 8 non-randomized studies, and 1 randomized crossover trial. Acute overall exercise showed a modest positive trend at lower dose levels; however, at a dose of 250 METs⋅min/week, the 95% credible interval included zero, indicating substantial uncertainty regarding whether a single bout of low-dose exercise produces a reliable increase in BDNF. For long-term overall exercise, posterior estimates generally favored a positive BDNF response as dose increased. The 95% credible interval first no longer included zero at 1300 METs⋅min/week, and estimates at 1700, 2000, 2300, and 2700 METs⋅min/week remained compatible with positive effects. However, these findings should be interpreted cautiously because the evidence base was limited and precision varied across dose levels. Clear differences were also observed across long-term exercise modalities. Multicomponent exercise showed the most favorable directional pattern across the 1700–2700 METs⋅min/week range, although several estimates were imprecise and the apparent advantage of this modality should be regarded as exploratory. By contrast, walking, cycling, and exercise combined with cognitive training all showed positive directional trends, but the 95% credible intervals included zero across dose levels, indicating substantial uncertainty regarding these modality-specific effects.

**Conclusion:**

The effects of exercise on BDNF in patients with stroke are jointly shaped by intervention duration, dose level, and exercise modality. Compared with acute exercise, long-term exercise at moderate-to-high doses, particularly multicomponent exercise, may be associated with a more favorable BDNF response. However, because the certainty of evidence was mostly moderate to low or very low and publication bias was detected, these findings should be considered preliminary and hypothesis-generating rather than definitive exercise prescription recommendations.

**Systematic review registration:**

https://www.crd.york.ac.uk/PROSPERO/view/CRD420261350793, identifier CRD420261350793.

## 1 Introduction

Stroke remains one of the leading causes of death and health loss worldwide. According to the Global Burden of Disease Study 2021, there were approximately 11.9 million incident cases, 93.8 million prevalent cases, and 7.3 million deaths attributable to stroke globally in 2021, ranking it as the third leading cause of death and the fourth leading cause of disability-adjusted life-year loss worldwide (GBD 2021 Stroke Risk Factor Collaborators, 2024). These figures underscore that stroke continues to represent a major global public health challenge (Owolabi et al., 2022; Tu et al., 2023). Beyond the high mortality risk in the acute phase, stroke often results in persistent motor, cognitive, and emotional impairments, as well as restricted social participation, thereby imposing a substantial burden on individuals, families, and healthcare systems (Hilkens et al., 2024). In recent years, the focus of stroke research has gradually extended from acute management to functional reconstruction during recovery. Accumulating evidence indicates that post-stroke functional outcomes are shaped not only by acute reperfusion therapy, but also by brain network reorganization associated with neuroplastic processes during the recovery stage (Delcamp et al., 2024). Identifying key neurobiological indicators that capture these processes has therefore become an important step in bridging clinical outcomes in stroke rehabilitation with mechanistic investigation.

Stroke care has long been centered on acute treatment and secondary prevention. However, because recovery for most patients primarily unfolds after hospital discharge, within the community and during the chronic stage, improvements in functional recovery, community reintegration, and long-term outcomes still depend heavily on sustained rehabilitation and transitional support (Hilkens et al., 2024; Reeves et al., 2023). Previous systematic reviews have shown that stroke survivors commonly experience substantial unmet rehabilitation needs during community-based recovery and long-term follow-up, suggesting that conventional medical pathways alone are insufficient to address the prolonged course of post-stroke recovery (Zawawi et al., 2020). Against this background, exercise has increasingly been regarded as a promising non-pharmacological strategy in stroke rehabilitation, not only because it can be embedded within routine rehabilitative practice, but also because it may extend beyond conventional functional training by influencing processes related to neuroplasticity (Feter et al., 2020; Talar et al., 2022). Given that higher serum brain-derived neurotrophic factor (BDNF) levels are independently associated with better prognosis after ischaemic stroke, and that BDNF is widely recognized as a marker linked to neuroplasticity, it may serve as an important bridging indicator between the clinical benefits of exercise and its underlying neurobiological mechanisms (Zhu et al., 2023).

Although the research pathway linking exercise, BDNF, and stroke recovery is beginning to take shape, current evidence has largely remained at the level of asking whether exercise is beneficial overall (Cakmak et al., 2024; Kayabinar et al., 2021). By contrast, far less is known about the differential effects associated with exercise modality, dose parameters, and intervention duration (Ashcroft et al., 2022). In a systematic review published in 2022, Ashcroft and colleagues explicitly noted that exercise parameters influence changes in BDNF among patients with stroke, and that high-intensity aerobic exercise may be more favorable than non-aerobic exercise for increasing circulating BDNF. However, the available studies were limited in number and characterized by considerable heterogeneity, precluding precise dose recommendations (Ashcroft et al., 2022). Similarly, a 2023 systematic review by Sivaramakrishnan and colleagues on the effects of acute aerobic exercise in individuals with chronic stroke concluded that the priming effects of a single bout of exercise on neurophysiological, molecular, and behavioral outcomes remain unclear, highlighting persistent inconsistency in the evidence regarding the immediate mechanisms of acute exercise (Sivaramakrishnan and Subramanian, 2023). In other words, what is currently lacking is not further repetition of the broad conclusion that exercise is beneficial, but a systematic quantification of how different exercise modalities, dose parameters, and temporal dimensions, acute versus long-term, relate to BDNF responses. This gap directly limits the development of precise exercise prescriptions for post-stroke rehabilitation.

Against this background, the present study systematically integrates the available evidence on the effects of exercise interventions on BDNF in patients with stroke and conducts dose-response analyses at both the acute and long-term levels. For acute exercise, the primary aim is to evaluate the relationship between overall exercise dose and improvement in BDNF. For long-term exercise, the analysis is extended by incorporating different exercise modalities in order to examine the differential effects of dose combinations on BDNF. Unlike conventional meta-analyses that focus primarily on pooled overall effects, this study applies a Bayesian dose-response meta-analytic framework that simultaneously incorporates dose information and comparative relationships among different long-term exercise modalities, making it better suited to identifying effect patterns under complex intervention conditions. The aim of this study is to clarify the potential differences between acute overall exercise dose and dose combinations across long-term exercise modalities in improving BDNF, thereby providing evidence to support biologically informed and precise exercise prescription in stroke rehabilitation. More broadly, this work seeks to offer more direct evidence for neuroplasticity-based precision exercise prescription after stroke and to provide a more layered basis for understanding the biological mechanisms through which exercise may facilitate stroke recovery.

## 2 Methodology

### 2.1 Data sources and search strategy

This study was conducted within the methodological framework of a systematic review and meta-analysis and was designed, screened, analysed, and reported in accordance with PRISMA recommendations. To enhance procedural transparency and ensure traceability of the review process, the protocol was prospectively registered in PROSPERO under the registration number CRD420261350793. No amendments to the registered protocol were made. Literature searches were performed in PubMed, Embase, Web of Science, the Cochrane Library, and APA PsycINFO, from database inception to 25 March 2026.

The search strategy was developed around the core concepts of the review question. Both controlled vocabulary and free-text terms were used, and multi-level Boolean combinations were applied to balance breadth of retrieval with accuracy of identification. As indexing systems, field structures, and syntax differ across databases, the search strings were locally adapted for each database within a unified conceptual framework to improve consistency and retrieval efficiency across sources. In addition to electronic database searching, backward citation tracking was undertaken to identify potentially missed studies. Specifically, the reference lists of relevant systematic reviews and narrative reviews published during the past 10 years were manually screened to broaden search coverage. All records were screened independently by two reviewers. Initial screening was based on titles and abstracts, followed by full-text eligibility assessment. Any disagreement arising during the selection process was resolved by consultation with a third reviewer, and final inclusion decisions were reached through discussion. The full search strategies and database-specific details are provided in Supplementary Material 1.

### 2.2 Eligibility criteria

Eligibility was determined using the PICOS framework, with assessment covering participants, interventions, comparators, outcomes, and study design.

First, with regard to participants, only human studies involving patients with stroke were considered eligible. Stroke status had to be established either by clinical diagnosis or by standardized assessment tools. To reduce confounding in the interpretation of findings, studies involving participants with Parkinson’s disease, dementia, or other central nervous system disorders were excluded. No restrictions were placed on sex, stroke subtype, or sociodemographic background. Studies conducted primarily in healthy individuals or other non-stroke populations were not considered eligible.

Second, with regard to interventions, studies were eligible if exercise training or physical activity constituted the core intervention. Acceptable forms included aerobic training, resistance exercise, walking-based training, mind–body exercise, multicomponent physical training, and combined exercise programmes incorporating virtual reality, cognitive tasks, or game-based interactive elements. Because the review aimed to extract and integrate a wide range of prescription characteristics, no prespecified thresholds were imposed for training frequency, exercise intensity, session duration, total intervention period, or interval structure.

Third, with regard to comparators, eligible control conditions included usual rehabilitation, conventional physical therapy, standard care, guidance on daily activities, and non-exercise interventions primarily based on cognitive training. Studies comparing two or more exercise programmes were also retained, provided that the intervention characteristics of each group could be clearly distinguished, thereby allowing subsequent comparative analyses across different exercise conditions.

For outcomes, brain-derived neurotrophic factor (BDNF) was specified as the core eligibility criterion. Only studies reporting BDNF outcomes were included, in order to ensure comparability and integrability of the primary outcome across studies.

With regard to study design, the overall number of exercise intervention studies in stroke reporting BDNF remains limited. Restricting inclusion to randomized controlled trials alone would therefore have substantially narrowed the available evidence base and reduced the ability to characterize dose-related information and pattern differences across acute and long-term interventions. Accordingly, provided that methodological quality could be adequately assessed, both randomized and non-randomized intervention studies were included to broaden evidence coverage and to allow a more comprehensive mapping of the current literature. All included studies were required to be full-text articles published in peer-reviewed journals and conducted in human participants.

Studies were excluded if they met any of the following conditions: they were study protocols, conference abstracts, theses, gray literature, or other unpublished materials; they used retrospective designs, case series designs, or observational cohort designs; or they failed to report the primary outcome data, or reported relevant outcomes in a form that did not allow extraction of raw data, calculation of effect sizes, or the required data transformation.

### 2.3 Data processing and extraction

Study identification and data extraction were performed independently by two reviewers in order to minimize the influence of subjective judgment on the results. The literature handling process involved sequential screening at the title, abstract, and full-text levels, followed by structured data extraction from the final set of included studies. If disagreements arose during study selection or data recording, the original article was rechecked and cross-verified. If consensus could still not be reached, a third reviewer reassessed the study and made the final decision. The extracted information primarily included the following domains: general study characteristics, including first author, year of publication, and country of conduct; participant characteristics, including sample size, mean age, and sex distribution; intervention-related information, including exercise content, frequency per week, total intervention duration, session length, corresponding metabolic equivalent values (METs), and the basis used for MET assignment; statistical results for the primary outcome at the relevant pre- and post-intervention time points; and details regarding outcome measurement methods.

For studies that did not directly report means and standard deviations, numerical conversion was performed using methods recommended by the Cochrane Handbook, based on available information such as sample size, median, range, or interquartile range. When only the standard error, confidence interval, or interquartile distance was reported, the corresponding statistical parameters required for subsequent analyses were derived using established formulae. If more than one instrument was used within the same study to assess the same outcome, the result with clearer measurement properties and greater suitability for cross-study synthesis was preferentially retained, in order to avoid multiple effect sizes from the same study contributing repeatedly to the pooled estimate (Chen et al., 2024).

### 2.4 Methodological quality assessment

To identify potential sources of bias arising from study design, implementation, and outcome reporting, risk-of-bias assessment was conducted using different tools according to study type. Randomized intervention studies were evaluated using the Revised Cochrane risk-of-bias tool for randomized trials (RoB 2.0) (Sterne et al., 2019), whereas non-randomized intervention studies were assessed using the Risk Of Bias In Non-randomized Studies of Interventions (ROBINS-I) tool (Sterne et al., 2016). Matching the assessment framework to the underlying study design allowed a more appropriate characterization of the quality profile of different forms of evidence.

Risk-of-bias assessment was carried out independently by two reviewers who had received training in systematic review methodology. Each study was judged separately under conditions of reviewer independence. Where discrepancies arose, the original report was re-examined and discussed until agreement was achieved. If disagreement persisted, a third reviewer conducted an additional evaluation and made the final judgment, thereby reducing the influence of individual subjectivity on the assessment process. RoB 2.0 was used primarily to assess the adequacy of the randomization process, deviations from intended interventions, completeness of outcome data, potential bias in outcome measurement, and selective reporting of results (Sterne et al., 2019). ROBINS-I was used primarily to evaluate bias arising from confounding, participant selection, intervention classification, deviations from intended interventions, missing data, outcome measurement, and selective reporting (Sterne et al., 2016). The results of these assessments were used as an important basis for describing the quality of the included evidence and for supporting the interpretation of findings and subsequent sensitivity analyses.

### 2.5 Model construction and computational procedures

The aim of this study was to determine whether a non-linear association exists between exercise exposure and changes in BDNF in patients with stroke. Accordingly, statistical analyses were conducted using a network dose-response meta-regression approach within a Bayesian random-effects framework, implemented as model-based network meta-analysis of dose-response (MBNMA; Mawdsley et al., 2016). Before parameter estimation, the intervention network was structurally examined to confirm that sufficient connectivity existed among intervention nodes to support quantitative analysis. This step also allowed assessment of the fundamental assumptions underlying network inference, including exchangeability across studies and overall consistency within the evidence structure (Wheeler et al., 2010).

The primary outcome was expressed as the standardized mean difference, with Hedges’ g applied as a small-sample correction. Corresponding 95% Bayesian credible intervals were calculated to represent the magnitude of BDNF change and its associated uncertainty across different levels of exercise exposure (Etzioni and Kadane, 1995). To identify the most appropriate functional form for the dose-response relationship, the observed data were first visually inspected in order to characterize the overall distributional pattern between dose and effect. Multiple candidate models were then fitted in sequence, including Emax functions, restricted cubic splines, quadratic polynomials, and other functional forms capable of capturing non-monotonic change (Pedder et al., 2019). Model selection was based on an integrated evaluation of the deviance information criterion, parameter complexity, residual behavior, and between-study heterogeneity. This comparison indicated that the quadratic polynomial model provided a more favorable balance between model fit and parameter stability and was therefore selected as the primary modeling approach for posterior estimation and dose-response inference (Evans, 2019).

For dose calculation, exercise programmes were first classified according to training content and delivery characteristics. Because all included studies reported sufficiently detailed exercise modalities and intervention characteristics, each exercise condition could be matched to the corresponding activity category in the 2024 Adult Compendium of Physical Activities. Standard MET values were then assigned according to the specific exercise modality rather than broad qualitative intensity labels alone. Weekly exercise dose was calculated as METs × session duration × weekly frequency and expressed as METs⋅min/week. When more than one Compendium category was potentially applicable, the closest match was selected according to the reported exercise content, delivery mode, and intensity description, and the assignment was checked by two reviewers. This approach allowed all included interventions to be converted into a common dose metric while preserving modality-specific exercise information (Herrmann et al., 2024). Weekly training duration was subsequently calculated from session length and frequency per week, and converted into a weekly dose metric expressed as METs⋅min/week by incorporating the corresponding MET value (Wasfy and Baggish, 2016). Because the reported range of energy exposure varied substantially across studies, direct inclusion of dose as a continuous variable could have increased numerical instability and the risk of disproportionate weighting during estimation. To improve comparability across studies and enhance model stability, dose values ranging from 100 to 3000 METs⋅min/week were therefore further grouped into a series of graded levels according to a geometric scaling principle (Higgins et al., 2012).

All statistical analyses were performed in R version 4.4.2. Non-linear network dose-response models were fitted using the MBNMAdose package, and posterior distributions were estimated by Markov chain Monte Carlo sampling. Dose-response curves and related visualizations were generated using ggplot2 to facilitate intuitive presentation of the modeled relationships and fitting results.

### 2.6 Evidence grading and assessment of result robustness

To evaluate the strength of evidence underlying the principal findings, the core outcomes were graded individually. All ratings were conducted independently by two reviewers. When discrepancies arose, the original study materials and assessment criteria were re-examined and discussed. If consensus could still not be reached, a third reviewer undertook an additional assessment and made the final judgment, thereby minimizing the influence of subjective variation on the rating process. The certainty of evidence was evaluated using the GRADE framework (Kavanagh, 2009). This approach considers five principal domains: risk of bias in the included studies, consistency of findings across studies, directness of the evidence in relation to the target question, precision of effect estimation, and the likelihood of publication bias. On the basis of an integrated appraisal across these domains, the certainty of evidence for the primary outcomes was classified as high, moderate, low, or very low, in order to reflect the degree of confidence that could be placed in the corresponding conclusions and the boundaries of their interpretation. The resulting evidence ratings were interpreted alongside the main analytical findings to support judgment of the robustness of the study conclusions and to provide an additional basis for subsequent interpretation and evaluation of potential clinical relevance.

## 3 Results

### 3.1 Literature search and study selection

**FIGURE 1:**
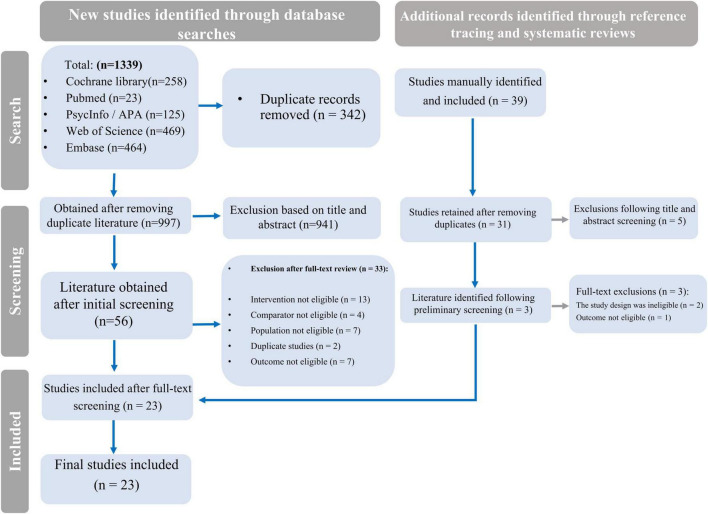
PRISMA flow diagram of the literature selection process. The flow diagram summarizes the identification, screening, eligibility assessment, and final inclusion of studies. A total of 1,339 records were identified through database searches, and 39 additional records were identified through reference tracing and previous systematic reviews. After duplicate removal, title and abstract screening, and full-text assessment, 23 studies were included in the final Bayesian network dose-response meta-analysis.

As of 25 March 2026, a systematic search of the Cochrane Library, PubMed, Embase, Web of Science, and APA PsycINFO identified 1339 records. The detailed search strategies are provided in Supplementary Table 1. After removal of duplicates, 997 records remained for title and abstract screening, of which 56 proceeded to full-text assessment. Following further evaluation against the prespecified inclusion and exclusion criteria, 23 studies identified through database searching were retained for inclusion. In parallel, 39 additional records were identified through reference tracking and screening of previous reviews. After deduplication, 31 records remained, of which 3 underwent full-text review. However, no additional eligible studies were identified through this process. A total of 23 studies were therefore included in the final analysis. The detailed study selection process is presented in Figure 1.

### 3.2 Characteristics of included studies

As shown in Supplementary File 2, a total of 23 studies involving 903 participants were ultimately included. Study designs comprised 14 randomized controlled trials, 8 non-randomized trials, and 1 randomized crossover trial. The included studies were published between 2014 and 2026 and were conducted across Egypt, Brazil, the United States, Canada, Australia, China, Russia, Indonesia, Switzerland, Spain, and Poland. Participants were predominantly middle-aged and older adults, with reported mean ages ranging from approximately 47.83–69.70 years. Most studies provided information on sex distribution, although this was not reported in several cases. The intervention modalities mainly included walking, cycle ergometer exercise, resistance exercise, multicomponent exercise, virtual reality training, and exercise combined with cognitive training. Some studies included multiple exercise arms to compare intervention effects across different intensities or programme types.

Exercise dose parameters varied substantially across studies. Reported MET values ranged from 2.8 to 8.8, intervention duration from 1 to 16 weeks, frequency from 1 to 7 sessions per week, and session length from 12 to 90 min per session. The included interventions covered both acute single-bout exercise and long-term structured training. All studies reported BDNF as the outcome measure.

### 3.3 Risk of bias and methodological quality assessment

The results of the risk-of-bias and methodological quality assessments are summarized in Supplementary Tables 2, 3. For the 15 randomized studies, the RoB 2.0 tool was applied across five domains: the randomization process, deviations from intended interventions, missing outcome data, measurement of the outcome, and selection of the reported result. Overall, most studies were judged as having some concerns, whereas only Hu et al. (2022) was rated as high risk. At the domain level, concerns were most commonly identified in relation to deviations from intended interventions. By contrast, performance was generally more favorable in the domains of missing outcome data and outcome measurement, although several individual studies were still judged as having high risk or some concerns in these areas. For the 8 non-randomized studies, methodological quality was assessed using the ROBINS-I tool, the JBI Critical Appraisal Checklist for Quasi-Experimental Studies, and the Newcastle–Ottawa Scale (NOS), as appropriate to the underlying study design. Of these, 4 studies were judged to be at serious risk of bias, whereas the remaining 4 were considered to be of moderate methodological quality. The principal methodological limitations among the non-randomized studies included the absence of random allocation, lack of an appropriate control condition, insufficient control for baseline confounding, and an elevated risk of selection bias. Overall, the methodological quality of the included studies was acceptable. However, most randomized studies still presented some degree of concern regarding bias, whereas the non-randomized studies showed more evident methodological limitations.

### 3.4 Assessment of local inconsistency

The node-splitting analysis indicated that local inconsistency across the network was generally low. In comparisons involving exercise modality and dose combinations, no significant inconsistency was detected between direct and indirect evidence, as shown in Supplementary Table 4. A similar pattern was observed for comparisons of overall exercise dose, with only a small number of low-dose comparisons showing some degree of inconsistency, as presented in Supplementary Table 5. Within the acute overall exercise dose network, none of the comparisons reached statistical significance, as shown in Supplementary Table 6. Taken together, these findings suggest that direct and indirect evidence were broadly compatible within the available network. However, this interpretation should remain cautious because the network was relatively sparse and several comparisons were informed by limited evidence.

### 3.5 Predicted effects of acute overall exercise in the low-dose range

**FIGURE 2:**
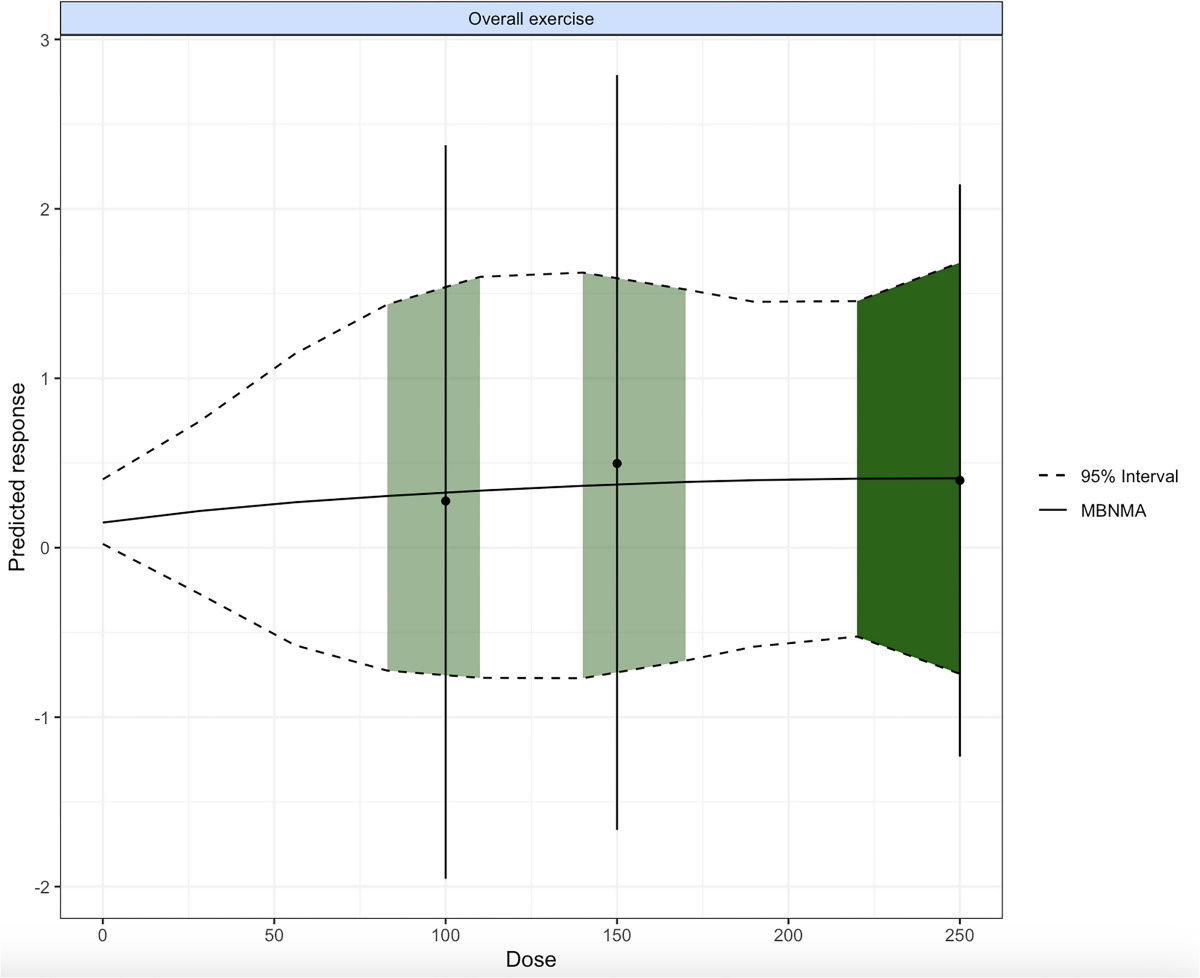
Dose-response relationship between overall exercise dose and predicted effect size in acute exercise. The solid black line represents the model-based network meta-analysis estimate, and the dashed lines represent the 95% Bayesian credible interval. Black points with vertical lines indicate predicted effects and corresponding uncertainty at specific dose levels. The shaded areas indicate the evaluated dose ranges. Within the acute low-dose range, the predicted effect showed a positive directional trend, but the 95% credible interval included zero, indicating substantial uncertainty regarding the effect of acute exercise on BDNF.

The predictive model for acute overall exercise in the low-dose range showed that, within the prespecified low-dose interval, the predicted effect at a dose level of 250 was 0.418, with a standard deviation of 0.579 and a median of 0.410. The corresponding 95% credible interval ranged from −0.744 to 1.683, as shown in Supplementary Table 9. Although the point estimate suggested a positive directional trend at this dose level, the 95% credible interval included zero, indicating substantial posterior uncertainty regarding the direction and magnitude of the effect. This finding suggests that the effect of acute overall exercise on BDNF remains uncertain within the low-dose range (Figure 2).

### 3.6 Predicted effects of long-term overall exercise dose

**FIGURE 3:**
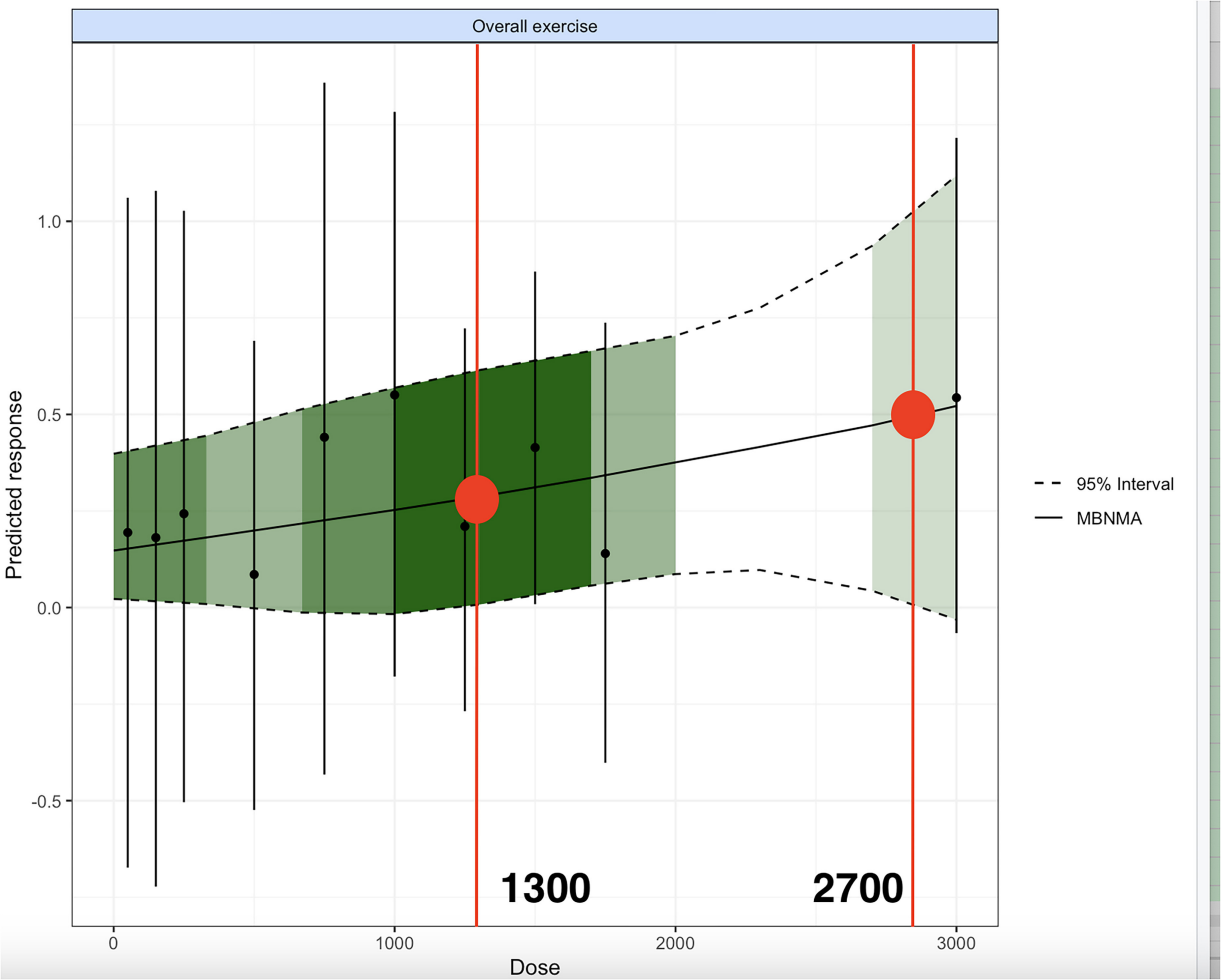
Dose-response relationship between overall exercise dose and predicted effect size in long-term exercise. The solid black line represents the model-based network meta-analysis estimate, and the dashed lines represent the 95% Bayesian credible interval. Black points with vertical lines indicate dose-specific predicted effects and corresponding uncertainty. The shaded areas indicate the evaluated dose ranges, and the red vertical markers highlight the dose range emphasized in the interpretation. The posterior estimates generally favored a positive BDNF response as dose increased, with clearer support for positive effects within the moderate-to-high dose range.

As shown in Supplementary Table 10, the predicted effect of long-term overall exercise generally increased with dose. Among the prespecified dose levels, the predicted effect at a dose of 1300 was 0.294, with a 95% credible interval of 0.007–0.615. This was the first dose level at which the 95% credible interval no longer included zero, suggesting posterior support for a positive effect at this dose level. Thereafter, the 95% credible intervals at dose levels of 1700, 2000, 2300, and 2700 METs⋅min/week also remained above zero, suggesting that the posterior estimates were compatible with positive effects within the moderate-to-high dose range. Nevertheless, the precision of these estimates varied across dose levels and should be interpreted in relation to the limited evidence base. At a dose of 2700, the predicted effect was 0.480. By contrast, the 95% credible intervals at dose levels of 330, 670, 1000, and 3000 METs⋅min/week included zero, indicating substantial uncertainty regarding the direction and magnitude of the predicted effects at these dose levels. Notably, although the point estimate at 3000 was relatively high (0.526), the interval estimate was wide and the evidence base corresponding to this dose level was limited. This result should therefore be interpreted with caution. Overall, the posterior estimates favored positive effects of long-term overall exercise primarily within the moderate-to-high dose range, whereas support for positive effects remained uncertain at lower and extremely high dose levels (Figure 3).

### 3.7 Predicted effects of different long-term exercise modalities and dose combinations

**FIGURE 4:**
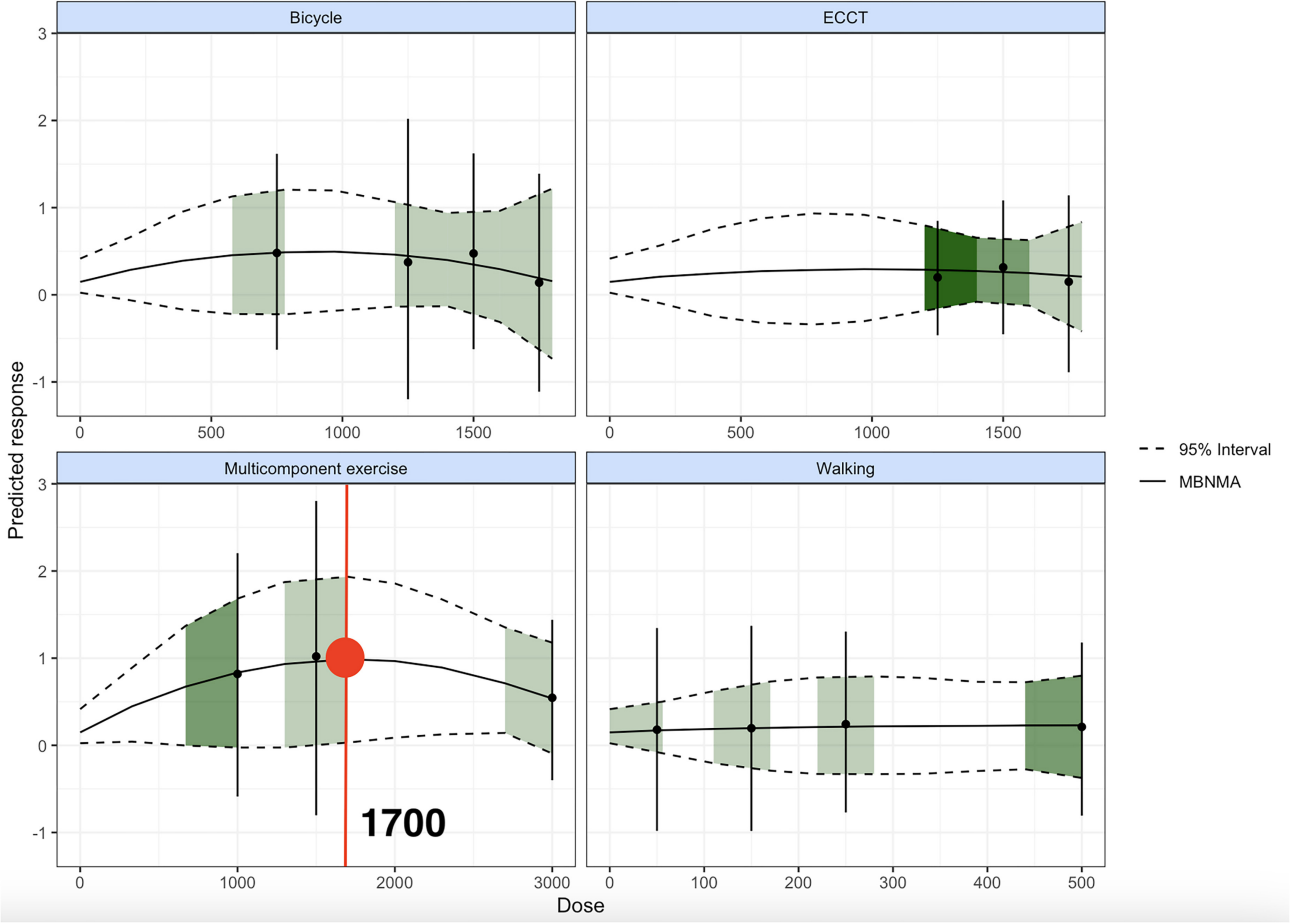
Dose-response relationship between modality-specific dose combinations and predicted effect size in long-term exercise. Separate panels show the estimated dose-response curves for bicycle exercise, exercise combined with cognitive training, multicomponent exercise, and walking. The solid black lines represent the model-based network meta-analysis estimates, and the dashed lines represent the 95% Bayesian credible intervals. Black points with vertical lines indicate predicted effects and corresponding uncertainty at specific dose levels. The red marker in the multicomponent exercise panel highlights the dose level emphasized in the interpretation. Multicomponent exercise showed the most favorable directional pattern within the moderate-to-high dose range, but the width of several credible intervals indicates that this finding should be interpreted cautiously.

Supplementary Table 11 shows that different long-term exercise modalities exhibited distinct patterns of predicted effects across dose levels. Multicomponent exercise showed the most favorable directional pattern within the moderate-to-high dose range, but the width of several credible intervals indicates that this finding remains imprecise. At a dose level of 1700, the predicted effect was 0.984, with a 95% credible interval of 0.031–1.930. The 95% credible intervals at 2000, 2300, and 2700 METs⋅min/week likewise did not include zero, indicating posterior support for positive effects at these dose levels. However, given the limited number of studies informing these estimates, the apparent advantage of multicomponent exercise should be interpreted as exploratory rather than confirmatory. By contrast, for cycling, walking, and exercise combined with cognitive training, the 95% credible intervals included zero at all dose levels, indicating substantial uncertainty regarding the predicted effects for these modalities. Overall, different long-term exercise modalities showed potentially different dose–response patterns, with multicomponent exercise presenting the most favorable directional pattern within the moderate-to-high dose range (Figure 4).

### 3.8 Assessment of evidence certainty

Preliminary GRADE assessment indicated that the certainty of evidence for the included exercise intervention studies was mainly distributed across the moderate-to-low range, as shown in Supplementary Tables 7, 8. In the GRADE framework, randomized studies began with a high level of certainty, but most were downgraded to moderate after consideration of risk-of-bias assessments, and a small number were further downgraded to low certainty. Non-randomized studies began with a low level of certainty and, after consideration of study design characteristics and methodological assessment, most remained at low or very low certainty. Overall, the certainty of the evidence included in this study was concentrated at moderate or lower levels, and the findings should therefore be interpreted in light of the underlying study designs and methodological context. Because the certainty of evidence was mainly moderate to low or very low, the dose-response estimates and modality rankings should not be interpreted as definitive clinical recommendations. Instead, they should be regarded as provisional evidence that requires confirmation in larger, high-quality randomized controlled trials with standardized exercise and BDNF assessment protocols.

### 3.9 Publication bias and small-study effects

**FIGURE 5:**
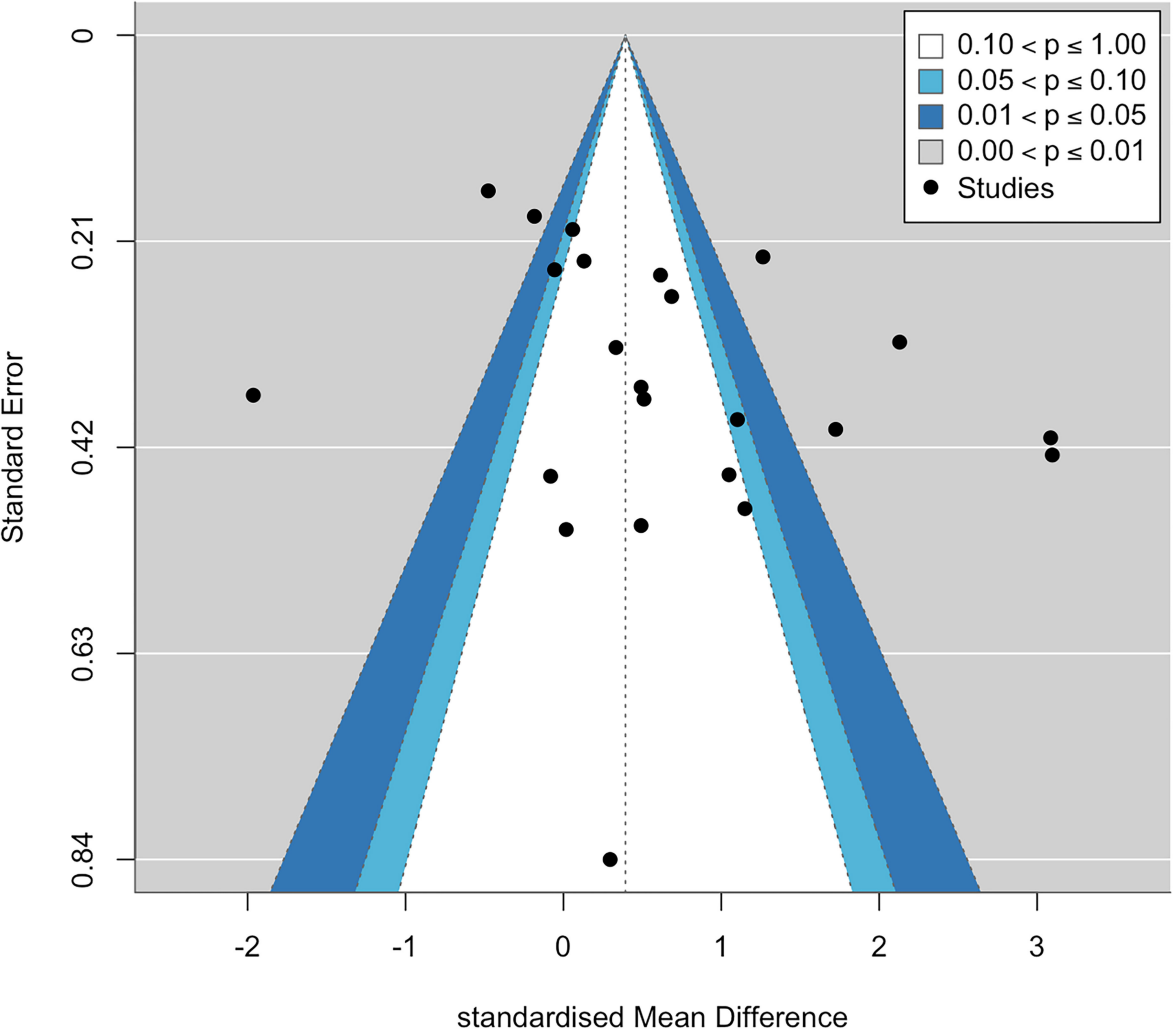
Contour-enhanced funnel plot for publication bias. Each black point represents an included effect size. The *x*-axis shows the standardized mean difference, and the *y*-axis shows the standard error. The contour regions correspond to different levels of statistical significance. Visual asymmetry in the distribution of studies suggests the possible presence of small-study effects or publication bias, which should be considered when interpreting the robustness of the pooled findings.

To examine the distributional characteristics of the included effect sizes, Egger’s regression test, a contour-enhanced funnel plot (Figure 5), and the trim-and-fill method were applied. Egger’s regression test reached statistical significance (*z* = 6.546, *p* = 0.040). The contour-enhanced funnel plot showed that some study points were distributed asymmetrically around the center. The trim-and-fill analysis estimated that 9 studies might be missing on the left side of the funnel plot (SE = 2.959). After imputation, the pooled effect size was −0.066, with a 95% confidence interval of −0.171 to 0.039 (*p* = 0.220). Taken together, the results of Egger’s regression test, the contour-enhanced funnel plot, and the trim-and-fill analysis all suggested a degree of asymmetry in the distribution of effect sizes across the included studies. After imputation, the adjusted pooled effect was close to zero and the interval estimate included zero, suggesting that small-study effects or publication bias may attenuate confidence in the observed positive findings. Therefore, although the main dose-response patterns provide useful preliminary signals, they should be interpreted cautiously in light of the detected funnel plot asymmetry and significant Egger’s test.

### 3.10 Robustness analysis

To evaluate the reliability of the dose-response findings, sensitivity analyses were conducted across three dimensions: model specification, study selection criteria, and leave-one-out perturbation. First, several non-linear dose-response functions were compared, and the quadratic function was selected as the primary analytical form based on both model fit and interpretability. Second, considering differences in study design and reporting across the included studies, the analyses were repeated by restricting the dataset to studies meeting stricter methodological criteria. The refitted dose-response curves, effect distribution patterns, and advantageous dose ranges were broadly consistent with the main analysis, with no material change in the overall conclusions. Finally, leave-one-out analyses were performed to examine the influence of each individual study on the pooled findings, with particular attention to studies contributing to the high-dose range. The results showed that excluding any single study did not materially alter the overall effect estimates or the interpretation of the dose ranges. Taken together, the dose-response relationships identified in the present study demonstrated good stability across multiple analytical conditions. Because all included studies reported sufficiently specific exercise modalities and intervention characteristics to allow mapping to the 2024 Adult Compendium of Physical Activities, no study was excluded because of an unconvertible exercise dose. Therefore, a sensitivity analysis excluding studies with insufficient exercise-form information was not applicable. Nevertheless, because the assigned MET values were based on standardized Compendium categories rather than direct patient-specific measurements, this issue was considered a source of uncertainty and is further addressed in the limitations section.

## 4 Discussion

### 4.1 Key findings

The most important finding of the present study does not lie in whether any single dose point showed posterior support for a positive effect, but rather in the potentially different response patterns observed across intervention durations. Specifically, acute overall exercise showed a favorable directional trend within the low-dose range, yet the corresponding interval estimates still crossed zero, indicating that the effects of a single exercise bout at lower exposure levels had not yet formed a stable and consistent signal of improvement. By contrast, long-term overall exercise displayed a clearer pattern of dose accumulation, with beneficial effects concentrated primarily within the moderate-to-high dose range. This suggests that sustained exercise stimulation may be more likely than short-lived exposure to produce detectable BDNF-related changes, although this interpretation remains constrained by the limited certainty of evidence.

More importantly, differences across long-term exercise modalities were not limited to simple variation in effect magnitude, but instead reflected a structural divergence in the dose–effect relationship itself. In particular, multicomponent exercise showed a more continuous and clearly defined pattern of improvement at moderate-to-high dose levels. Taken together, these findings indicate that the present study does not merely address the linear question of whether exercise is effective, but rather highlights a more complex process in which exercise effects are jointly shaped by intervention duration, cumulative dose, and training modality. In other words, the beneficial effects of exercise do not appear to arise uniformly across all doses and all forms of intervention, but are more likely to depend on a sufficient stimulus threshold, sustained exposure, and synergistic interactions among multiple training components. In this sense, the present study extends current understanding of the heterogeneity of exercise effects and provides a basis for shifting the discussion from whether an effect exists to the conditions under which an effect is more likely to emerge.

### 4.2 The effects of acute low-dose overall exercise may represent an early stage of response emergence

In the present study, acute overall exercise yielded a positive point estimate within the low-dose range, but the 95% credible interval included zero, indicating that the reliability of this effect remains uncertain. This finding first suggests that the effects of acute exercise may be better understood as a response pattern that can be transiently activated, but does not necessarily manifest as a stable and externally detectable improvement (Chang et al., 2025). High-level evidence has shown that acute exercise generally exerts small-to-moderate beneficial effects on cognitive function, but these effects are not strong or invariant and instead retain considerable scope for fluctuation (Chang et al., 2012). One systematic review reported that the priming effects of a single bout of acute aerobic exercise on neurophysiological, molecular, and behavioral outcomes in individuals with chronic stroke remain inconclusive, and that for molecular markers such as BDNF, only limited evidence of modulation is currently available. This suggests that evidence supporting the immediate induction of neuroplasticity-related responses after stroke by acute exercise remains insufficient (Sivaramakrishnan and Subramanian, 2023).

Against this background, the finding in the present study that the low-dose range showed a favorable direction but unstable evidence more likely indicates that low-dose acute exercise already has the potential to induce changes in BDNF, but that the stimulus intensity or overall exposure may still be insufficient to generate an immediate response that is consistent and reproducible (Ashcroft et al., 2022; Mcsween et al., 2019). Given that higher serum BDNF levels are associated with better prognosis after ischaemic stroke, and that BDNF is regarded as an important molecular basis of post-stroke neuroplasticity, the absence of a stable increase in the low-dose acute range should not be interpreted as evidence that exercise lacks rehabilitative value. Rather, it suggests that exercise interventions targeting BDNF after stroke may still require further optimization in terms of stimulus parameters and intervention configuration (Mang et al., 2013; Sivaramakrishnan and Subramanian, 2023).

### 4.3 Dose accumulation in long-term exercise and differential responses across exercise modalities

Unlike acute exercise, which relies primarily on short-term physiological activation, long-term exercise is more likely to promote post-stroke neuroplastic reconstruction through repeated stimulation and gradual accumulation. Its effects therefore tend not to emerge at low levels of exposure, but instead become more stable only after a certain training threshold has been reached (Donnellan-Fernandez et al., 2022; Limaye et al., 2021). In the present study, the positive predicted effects of long-term overall exercise were concentrated mainly within the moderate-to-high dose range, whereas evidence remained relatively limited at lower and extremely high doses. This pattern suggests that improvement in BDNF does not follow a simple linear increase, but may instead reflect a dynamic process characterized by cumulative exposure, threshold triggering, and subsequent plateau-like fluctuation. Previous reviews have indicated that aerobic training can promote post-stroke increases in BDNF and enhance neuroplasticity, but the magnitude of this response is strongly influenced by training parameters, intervention duration, and individual condition. Current evidence overall supports the view that training performed at moderate to relatively high intensity over several weeks is more likely to induce detectable molecular change (Ashcroft et al., 2022; Teasell et al., 2024).

More importantly, differences across long-term exercise modalities appear to involve not only variation in effect magnitude, but also structural divergence in the pathways through which those effects are produced. In the present study, multicomponent exercise showed a more continuous pattern of improvement within the moderate-to-high dose range, a finding that is mechanistically plausible. Compared with single-modality training, multicomponent exercise often integrates aerobic loading, strength stimulation, balance control, and task-switching demands within the same programme. Such training may therefore provide richer input across multiple levels, including cardiorespiratory adaptation, sensorimotor integration, and higher-order executive regulation. This provides a plausible explanation for the observed directional advantage of multicomponent exercise, but the present evidence is insufficient to confirm that this modality is superior to other exercise forms (Hill et al., 2023; Li et al., 2024; Skidmore et al., 2023). Existing studies and perspective papers have likewise suggested that, if exercise is to more effectively support post-stroke recovery, attention should not be limited to how much training is delivered, but should also consider whether the intervention creates a composite stimulus environment more conducive to neural network reconstruction (Kwakkel et al., 2023; Mang et al., 2013). This may also explain why walking or cycling alone can produce directional benefit, yet may not show molecular responses that are as stable as those observed with multicomponent training under current evidence conditions.

### 4.4 Implications and limitations

The present study has several implications for post-stroke rehabilitation research and practice. First, its contribution lies not simply in confirming that exercise may influence BDNF in patients with stroke, but in showing that this relationship is shaped by both the temporal dimension of intervention and the structure of exercise exposure. By distinguishing between acute and long-term exercise, and by further examining dose-related variation across different long-term exercise modalities, this study moves beyond a binary interpretation of exercise effectiveness and provides a more differentiated evidence base for biologically informed exercise prescription. In particular, the finding that acute low-dose exercise showed only an unstable positive trend, whereas long-term exercise produced clearer effects within the moderate-to-high dose range, suggests that the design of post-stroke exercise programmes should take account not only of whether exercise is prescribed, but also of how long it is sustained, at what dose it is delivered, and in what form it is organized.

Second, the present findings may also have potential value for the interpretation of exercise-induced neuroplasticity in stroke recovery. Because higher circulating BDNF levels have been associated with better prognosis after ischaemic stroke, the dose-related patterns observed here provide a useful perspective for linking intervention design with possible biological responsiveness. The results suggest that BDNF-related exercise effects after stroke are unlikely to arise uniformly across all conditions, and may instead depend on a sufficient threshold of repeated stimulation. This point is particularly relevant for long-term multicomponent exercise, which showed a more continuous positive trend within the moderate-to-high dose range. Although this pattern should not be overstated, it supports the view that exercise interventions integrating multiple training demands may offer a more favorable stimulus environment for neuroplastic adaptation than single-modality training alone.

Several limitations should nevertheless be considered. First, the number of eligible studies was limited overall, and evidence was especially sparse within certain dose ranges for acute exercise and for several long-term exercise modalities. This may have reduced the stability of the modeled dose-response curves and widened the uncertainty around some interval estimates. Second, substantial heterogeneity remained across studies in intervention protocol, training duration, participant characteristics, timing of BDNF collection, and laboratory assay procedures. These differences may all influence the observed BDNF response and complicate direct comparison across studies. Third, the present analysis was based primarily on peripheral circulating BDNF. Although this marker is widely used in exercise and stroke research as an index related to neuroplasticity, it cannot be assumed to fully represent biological processes within the central nervous system. Caution is therefore needed when extending these findings to mechanistic interpretations of neural recovery.

In addition, substantial clinical and methodological heterogeneity should be emphasized. The included studies differed in stroke phase, time since stroke, lesion characteristics, comorbidities, exercise protocols, and BDNF sampling procedures, all of which may influence BDNF responsiveness and partly explain the uncertainty of several estimates. Although subgroup analyses by stroke phase, lesion location, medication use, and BDNF sampling time would have been clinically informative, they were not feasible because these variables were inconsistently reported and the number of studies within each subgroup was limited. Another limitation concerns exercise dose estimation. Although all included studies reported sufficiently specific exercise modalities and intervention characteristics to permit conversion into METs⋅min/week using the 2024 Adult Compendium of Physical Activities, the assigned MET values were standardized Compendium-based estimates rather than direct measurements of patient-specific energy expenditure. Because energy cost and physiological load may differ between patients with stroke and the general adult populations represented in the Compendium, the identified dose ranges should be interpreted as approximate exposure categories rather than precise prescription thresholds. In addition, trial registries such as ClinicalTrials.gov and the WHO International Clinical Trials Registry Platform were not searched; thus, unpublished or ongoing trials may not have been fully captured, potentially contributing to publication bias. Finally, peripheral BDNF should be interpreted as a surrogate biomarker rather than a direct measure of central neuroplasticity. Although circulating BDNF is widely used in exercise and stroke research, peripheral changes do not necessarily mirror BDNF activity within the central nervous system. Accordingly, these findings should not be interpreted as direct evidence that a specific exercise dose or modality produces superior neural repair. The presence of funnel plot asymmetry, a significant Egger’s test, and trim-and-fill results suggesting potentially missing studies, together with the predominance of moderate-to-low or very-low certainty evidence, indicates that the current findings should be considered preliminary and hypothesis-generating. Future trials should adopt randomized designs with adequate sample sizes, directly measured exercise intensity, standardized BDNF sampling time points, and harmonized reporting of stroke-related clinical characteristics.

A further point is that the apparent advantage of long-term multicomponent exercise should be interpreted as a directional finding rather than a definitive conclusion. The current evidence suggests a more favorable trend for this modality within a specific dose range, but the underlying evidence base remains limited and uneven. Future studies with more rigorous designs, clearer dose reporting, and better integration of molecular, neurophysiological, and functional outcomes will be needed to determine more precisely which combinations of modality, dose, and duration are most conducive to post-stroke recovery.

## 5 Conclusion

Using a Bayesian dose-response meta-analytic framework, this study systematically evaluated the effects of acute and long-term exercise interventions on BDNF in patients with stroke. Acute overall exercise showed a positive directional trend within the low-dose range, but the 95% credible interval included zero, indicating substantial uncertainty regarding the reliability of this effect. For long-term overall exercise, posterior estimates tended to favor positive BDNF responses within the moderate-to-high dose range, particularly between 1300 and 2700 METs⋅min/week. However, the precision of these estimates varied and the underlying evidence base was limited.

Across long-term exercise modalities, multicomponent exercise showed the most favorable directional pattern within the moderate-to-high dose range. Nevertheless, this apparent advantage should be interpreted as exploratory rather than confirmatory because several estimates were imprecise, the certainty of evidence was mostly moderate to low or very low, and publication bias was detected. Overall, the present findings suggest that BDNF-related responses to exercise after stroke may be influenced by intervention duration, dose level, and exercise modality, but they do not yet provide definitive exercise prescription thresholds. Larger, high-quality randomized controlled trials with directly measured exercise intensity, standardized BDNF sampling protocols, and detailed reporting of stroke-related clinical characteristics are needed to confirm these dose-response patterns.

## Data Availability

The original contributions presented in this study are included in the article/Supplementary material, further inquiries can be directed to the corresponding author.
