# Supplementary material for: Effects of acute and long-term exercise modalities and doses on brain-derived neurotrophic factor in patients with stroke: a Bayesian network meta-analysis
Source: Front Syst Neurosci. 2026 Jul 24;20:1905191. doi: 10.3389/fnsys.2026.1905191 (PMC13447430; doi:10.3389/fnsys.2026.1905191)
Supplement: Supplementary file 1 [file Table_1.docx]

| Supplementary Table 1. Detailed search strategies for each database | | | |
| --- | --- | --- | --- |
| **Database** | **Search Terms for Population** | **Search Terms for Intervention** | **Search Terms for Outcomes** |
| Cochrane Library (limited to trials, N=258) | stroke: ti, ab, kw | aerobic OR balance training OR virtual reality OR strength OR yoga OR locomotion OR endurance OR tai chi OR functional training OR walk OR sport OR conditioning OR kinesiotherapy OR motor control OR pilates OR resistance OR activity OR hydrotherapy OR run OR posture OR high intensity interval OR dance OR neuromuscular OR water sports OR martial art OR stretch OR athletic OR physical OR swim OR core control OR mind-body exercise OR stability OR exercise OR isometric OR sprint OR activities OR sports combined training OR train: ti, ab, kw | brain-derived neurotrophic factor OR BDNF: ti, ab, kw |
| Web of Science (limited to Article, N = 469) | AB=(stroke) | AB=(aerobic OR balance training OR virtual reality OR strength OR yoga OR locomotion OR endurance OR tai chi OR functional training OR walk OR sport OR conditioning OR kinesiotherapy OR motor control OR pilates OR resistance OR activity OR hydrotherapy OR run OR posture OR high intensity interval OR dance OR neuromuscular OR water sports OR martial art OR stretch OR athletic OR physical OR swim OR core control OR mind-body exercise OR stability OR exercise OR isometric OR sprint OR activities OR sports combined training OR train: ti, ab, kw) | AB=(brain-derived neurotrophic factor OR BDNF) |
| PubMed (limited to RCTs OR Clinical Trial, N = 23) | stroke[Title/Abstract] | aerobic[Title/Abstract] OR balance training[Title/Abstract] OR virtual reality[Title/Abstract] OR strength[Title/Abstract] OR yoga[Title/Abstract] OR locomotion[Title/Abstract] OR endurance[Title/Abstract] OR tai chi[Title/Abstract] OR functional training[Title/Abstract] OR walk[Title/Abstract] OR sport[Title/Abstract] OR conditioning[Title/Abstract] OR kinesiotherapy[Title/Abstract] OR motor control[Title/Abstract] OR pilates[Title/Abstract] OR resistance[Title/Abstract] OR activity[Title/Abstract] OR hydrotherapy[Title/Abstract] OR run[Title/Abstract] OR posture[Title/Abstract] OR high intensity interval[Title/Abstract] OR dance[Title/Abstract] OR neuromuscular[Title/Abstract] OR water sports[Title/Abstract] OR martial art[Title/Abstract] OR stretch[Title/Abstract] OR athletic[Title/Abstract] OR physical[Title/Abstract] OR swim[Title/Abstract] OR core control[Title/Abstract] OR mind-body exercise[Title/Abstract] OR stability[Title/Abstract] OR exercise[Title/Abstract] OR isometric[Title/Abstract] OR sprint[Title/Abstract] OR activities[Title/Abstract] OR sports combined training[Title/Abstract] OR train[Title/Abstract] | brain-derived neurotrophic factor[Title/Abstract] OR BDNF[Title/Abstract] |
| Embase (limited to controlled study, N = 464) | stroke:ti,ab,kw | aerobic:ti,ab,kw OR 'balance training':ti,ab,kw OR 'virtual reality':ti,ab,kw OR strength:ti,ab,kw OR yoga:ti,ab,kw OR locomotion:ti,ab,kw OR endurance:ti,ab,kw OR 'tai chi':ti,ab,kw OR 'functional training':ti,ab,kw OR walk:ti,ab,kw OR sport:ti,ab,kw OR conditioning:ti,ab,kw OR kinesiotherapy:ti,ab,kw OR 'motor control':ti,ab,kw OR pilates:ti,ab,kw OR resistance:ti,ab,kw OR activity:ti,ab,kw OR hydrotherapy:ti,ab,kw OR run:ti,ab,kw OR posture:ti,ab,kw OR 'high intensity interval':ti,ab,kw OR dance:ti,ab,kw OR neuromuscular:ti,ab,kw OR 'water sports':ti,ab,kw OR 'martial art':ti,ab,kw OR stretch:ti,ab,kw OR athletic:ti,ab,kw OR physical:ti,ab,kw OR swim:ti,ab,kw OR 'core control':ti,ab,kw OR 'mind-body exercise':ti,ab,kw OR stability:ti,ab,kw OR exercise:ti,ab,kw OR isometric:ti,ab,kw OR sprint:ti,ab,kw OR activities:ti,ab,kw OR 'sports combined training':ti,ab,kw OR train:ti,ab,kw | 'brain-derived neurotrophic factor':ti,ab,kw OR bdnf:ti,ab,kw |
| PsycInfo / APA (N = 125) | Abstract: stroke | Abstract: aerobic OR balance training OR virtual reality OR strength OR yoga OR locomotion OR endurance OR tai chi OR functional training OR walk OR sport OR conditioning OR kinesiotherapy OR motor control OR pilates OR resistance OR activity OR hydrotherapy OR run OR posture OR high intensity interval OR dance OR neuromuscular OR water sports OR martial art OR stretch OR athletic OR physical OR swim OR core control OR mind-body exercise OR stability OR exercise OR isometric OR sprint OR activities OR sports combined training OR train | Abstract: brain-derived neurotrophic factor OR BDNF |

Supplementary Table 2. Risk of bias assessment of randomized studies using RoB 2.0

| **Study** | **Randomization process** | **Deviations from intended interventions** | **Missing outcome data** | **Measurement of the outcome** | **Selection of the reported result** | **Overall risk** |
| --- | --- | --- | --- | --- | --- | --- |
| Valkenborghs (2019, Australia) | Low risk | Some concerns | Low risk | Low risk | Some concerns | Some concerns |
| Ploughman (2019, Canada) | Low risk | Some concerns | Low risk | Low risk | Low risk | Some concerns |
| Boyne (2020, America) | Some concerns | Some concerns | Low risk | Some concerns | Low risk | Some concerns |
| Hsu (2020, Taiwan, China) | Low risk | Some concerns | Low risk | Low risk | Low risk | Some concerns |
| Rahayu (2020, Indonesia) | Some concerns | Some concerns | Low risk | Low risk | Low risk | Some concerns |
| Du (2021, China) | Some concerns | Some concerns | Low risk | Some concerns | Low risk | Some concerns |
| Mackay (2021, Australia) | Some concerns | Some concerns | Low risk | Some concerns | Low risk | Some concerns |
| Hu (2022, China) | Some concerns | Some concerns | Low risk | Some concerns | Some concerns | High risk |
| Huang (2022, Taiwan) | Some concerns | Some concerns | Low risk | Low risk | Low risk | Some concerns |
| Maguire (2023, Switzerland) | Low risk | Some concerns | High risk | Some concerns | Low risk | Some concerns |
| Bermudo-Gallaguet (2025, Spain) | Low risk | Some concerns | Low risk | Low risk | Low risk | **Some concerns** |
| De Las Heras (2025, Canada) | Some concerns | Some concerns | Some concerns | Low risk | Low risk | **Some concerns** |
| García-Salazar (2025, Brazil) | Some concerns | Some concerns | Low risk | Low risk | Low risk | **Some concerns** |
| Górna (2025, Poland) | Some concerns | Some concerns | High risk | Low risk | Low risk | **Some concerns** |
| Mackay (2026, Australia) | Low risk | Some concerns | Some concerns | Low risk | Low risk | **Some concerns** |

Supplementary Table 3. Methodological appraisal, overall judgement, and key concerns of non-randomized studies

| **Study** | **Appraisal tool** | **Overall judgement** | **Key concern / remark** |
| --- | --- | --- | --- |
| El-Tamawy (2014, Egypt) | ROBINS-I | Serious risk of bias | Non-randomized design with potential confounding and selection bias. |
| de Morais (2017, Brazil) | JBI Critical Appraisal Checklist for Quasi-Experimental Studies | Moderate methodological quality | No control group, limiting causal interpretation. |
| Charalambous (2018, America) | ROBINS-I | Serious risk of bias | Non-randomized group comparison with possible confounding. |
| Harnish (2018, America) | JBI Critical Appraisal Checklist for Quasi-Experimental Studies | Moderate methodological quality | Single-subject non-randomized design with limited external validity. |
| King (2019, Canada) | JBI Critical Appraisal Checklist for Quasi-Experimental Studies | Moderate methodological quality | Single-group pre–post non-randomized exercise study without a parallel control group, limiting causal interpretation. |
| Ryan (2019, America) | JBI Critical Appraisal Checklist for Quasi-Experimental Studies | Moderate methodological quality | No randomized allocation or parallel control group. |
| Koroleva (2020, Russia) | ROBINS-I | Serious risk of bias | Non-randomized allocation with possible baseline imbalance. |
| Shi (2023, China) | ROBINS-I | Serious risk of bias | Retrospective non-randomized design with possible bias and residual confounding. |

Note: Overall judgements were reported according to the original recommendations of each appraisal tool and should not be directly compared across different tools. Abbreviations: ROBINS-I, Risk of Bias in Non-randomized Studies - of Interventions; JBI, Joanna Briggs Institute; NOS, Newcastle-Ottawa Scale.

Supplementary Table 4. Node-splitting analysis for local inconsistency across exercise modalities and dose combinations

| **Comparison** | **Evidence source** | **p-value** | **Median** | **2.50%** | **97.50%** |
| --- | --- | --- | --- | --- | --- |
| Walking_500 vs Multicomponent exercise_1000 | direct | 0.333 | -0.606 | -1.205 | -0.03 |
|  | indirect |  | -0.074 | -0.614 | 0.48 |
|  | MBNMA |  | -0.304 | -0.719 | 0.104 |
| Multicomponent exercise_1500 vs Multicomponent exercise_1000 | direct | 0.18 | 0.203 | -0.733 | 1.142 |
|  | indirect |  | 0.079 | -0.01 | 0.169 |
|  | MBNMA |  | 0.078 | -0.013 | 0.166 |
| Walking_500 vs Placebo_0 | direct | 0.451 | 0.069 | -0.475 | 0.62 |
|  | indirect |  | -0.356 | -0.949 | 0.23 |
|  | MBNMA |  | -0.142 | -0.569 | 0.234 |
| Walking_250 vs Placebo_0 | direct | 0.431 | 0.099 | -0.598 | 0.746 |
|  | indirect |  | -0.087 | -0.295 | 0.125 |
|  | MBNMA |  | -0.071 | -0.284 | 0.117 |
| Walking_150 vs Placebo_0 | direct | 0.258 | -0.008 | -0.87 | 0.844 |
|  | indirect |  | -0.046 | -0.169 | 0.075 |
|  | MBNMA |  | -0.043 | -0.171 | 0.07 |
| Walking_50 vs Placebo_0 | direct | 0.113 | 0.016 | -0.801 | 0.802 |
|  | indirect |  | -0.014 | -0.052 | 0.028 |
|  | MBNMA |  | -0.014 | -0.057 | 0.023 |
| Multicomponent exercise_3000 vs Placebo_0 | direct | 0.187 | 0.364 | -0.179 | 0.924 |
|  | indirect |  | 1.99 | -0.225 | 4.037 |
|  | MBNMA |  | 0.465 | -0.079 | 0.997 |
| ECCT_1750 vs Placebo_0 | direct | 0.705 | -0.009 | -0.573 | 0.546 |
|  | indirect |  | 0.145 | -0.243 | 0.544 |
|  | MBNMA |  | 0.094 | -0.227 | 0.427 |
| ECCT_1500 vs Placebo_0 | direct | 0.625 | 0.177 | -0.25 | 0.579 |
|  | indirect |  | -0.016 | -0.347 | 0.346 |
|  | MBNMA |  | 0.081 | -0.194 | 0.366 |
| ECCT_1250 vs Placebo_0 | direct | 0.698 | 0.036 | -0.443 | 0.521 |
|  | indirect |  | 0.075 | -0.192 | 0.348 |
|  | MBNMA |  | 0.067 | -0.162 | 0.305 |
| Bicycle_1750 vs Placebo_0 | direct | 0.644 | -0.014 | -1.032 | 0.921 |
|  | indirect |  | 0.39 | -0.395 | 1.119 |
|  | MBNMA |  | 0.27 | -0.345 | 0.821 |
| Bicycle_1500 vs Placebo_0 | direct | 0.834 | 0.295 | -0.456 | 1.06 |
|  | indirect |  | 0.152 | -0.508 | 0.853 |
|  | MBNMA |  | 0.232 | -0.295 | 0.703 |
| Bicycle_1250 vs Placebo_0 | direct | 0.477 | 0.183 | -1.063 | 1.605 |
|  | indirect |  | 0.162 | -0.271 | 0.582 |
|  | MBNMA |  | 0.193 | -0.246 | 0.586 |
| Bicycle_750 vs Placebo_0 | direct | 0.473 | 0.301 | -0.493 | 1.084 |
|  | indirect |  | 0.093 | -0.18 | 0.352 |
|  | MBNMA |  | 0.116 | -0.148 | 0.352 |

Supplementary Table 5. Node-splitting analysis of local inconsistency across total exercise dose comparisons

| **Comparison** | **Evidence source** | **p-value** | **Median** | **2.50%** | **97.50%** |
| --- | --- | --- | --- | --- | --- |
| Overall exercise_1500 vs Overall exercise_1000 | direct | 0.138 | 0.206 | -0.666 | 1.07 |
|  | indirect |  | 0.055 | -0.003 | 0.112 |
|  | MBNMA |  | 0.054 | -0.002 | 0.115 |
| Overall exercise_3000 vs Placebo_0 | direct | 0.817 | 0.383 | -0.158 | 0.942 |
|  | indirect |  | 0.278 | -0.199 | 0.743 |
|  | MBNMA |  | 0.324 | -0.01 | 0.689 |
| Overall exercise_1750 vs Placebo_0 | direct | 0.463 | 0.005 | -0.482 | 0.46 |
|  | indirect |  | 0.239 | 0.003 | 0.479 |
|  | MBNMA |  | 0.189 | -0.006 | 0.402 |
| Overall exercise_1250 vs Placebo_0 | direct | 0.48 | 0.045 | -0.374 | 0.506 |
|  | indirect |  | 0.146 | -0.014 | 0.301 |
|  | MBNMA |  | 0.135 | -0.004 | 0.287 |
| Overall exercise_750 vs Placebo_0 | direct | 0.204 | 0.301 | -0.508 | 1.068 |
|  | indirect |  | 0.079 | -0.013 | 0.165 |
|  | MBNMA |  | 0.081 | -0.002 | 0.172 |
| Overall exercise_250 vs Placebo_0 | direct | 0.102 | 0.092 | -0.589 | 0.827 |
|  | indirect |  | 0.027 | -0.003 | 0.056 |
|  | MBNMA |  | 0.027 | -0.001 | 0.057 |
| Overall exercise_150 vs Placebo_0 | direct | 0.051 | 0.057 | -0.836 | 0.846 |
|  | indirect |  | 0.016 | -0.003 | 0.032 |
|  | MBNMA |  | 0.016 | 0 | 0.034 |
| Overall exercise_50 vs Placebo_0 | direct | 0.022 | -0.001 | -0.766 | 0.799 |
|  | indirect |  | 0.005 | 0 | 0.011 |
|  | MBNMA |  | 0.005 | 0 | 0.011 |

Supplementary Table 6. Node-splitting analysis of local inconsistency for comparisons of acute total exercise dose versus placebo

| **Comparison** | **Evidence source** | **p-value** | **Median** | **0.025** | **0.975** |
| --- | --- | --- | --- | --- | --- |
| Overall exercise_250 vs Placebo_0 | direct | 0.78 | 0.242 | -0.83 | 1.318 |
|  | indirect |  | 0.375 | -1.452 | 2.46 |
|  | MBNMA |  | 0.265 | -0.418 | 0.981 |
| Overall exercise_150 vs Placebo_0 | direct | 0.63 | 0.3 | -1.311 | 1.698 |
|  | indirect |  | 0.141 | -0.494 | 0.827 |
|  | MBNMA |  | 0.159 | -0.251 | 0.589 |
| Overall exercise_100 vs Placebo_0 | direct | 0.582 | 0.094 | -1.472 | 1.649 |
|  | indirect |  | 0.108 | -0.302 | 0.559 |
|  | MBNMA |  | 0.106 | -0.167 | 0.392 |

Supplementary Table 7. Preliminary GRADE assessment for randomized studies

| **Study** | **Study design** | **Initial certainty** | **Main reason for downgrading** | **Suggested GRADE certainty** |
| --- | --- | --- | --- | --- |
| Valkenborghs (2019, Australia) | Randomized controlled trial | High | Some concerns in risk of bias | Moderate |
| Ploughman (2019, Canada) | Randomized controlled trial | High | Some concerns in risk of bias | Moderate |
| Boyne (2020, America) | Randomized crossover trial | High | Some concerns related to crossover design and risk of bias | Moderate |
| Hsu (2020, Taiwan, China) | Randomized controlled trial | High | Some concerns in risk of bias | Moderate |
| Rahayu (2020, Indonesia) | Randomized controlled trial | High | Some concerns in risk of bias | Moderate |
| Du (2021, China) | Randomized crossover / repeated-measures trial | High | Some concerns related to crossover design and risk of bias | Moderate |
| Mackay (2021, Australia) | Randomized crossover trial | High | Some concerns related to crossover design and outcome assessment | Moderate |
| Hu (2022, China) | Randomized controlled trial | High | High risk of bias | Low |
| Huang (2022, Taiwan) | Assessor-blinded randomized controlled trial | High | Some concerns in risk of bias | Moderate |
| Maguire (2023, Switzerland) | Randomized controlled feasibility study | High | Some concerns in risk of bias, especially missing outcome data | Moderate |
| Bermudo-Gallaguet (2025, Spain) | Single-blind randomized controlled trial | High | Some concerns in risk of bias | Moderate |
| De Las Heras (2025, Canada) | Randomized controlled trial | High | Some concerns in risk of bias and missing outcome data | Moderate |
| García-Salazar (2025, Brazil) | Pilot randomized controlled trial | High | Some concerns in risk of bias | Moderate |
| Górna (2025, Poland) | Randomized clinical trial | High | Some concerns in risk of bias, especially missing outcome data | Moderate |
| Mackay (2026, Australia) | Randomized controlled trial | High | Some concerns in risk of bias | Moderate |

Notes: Randomized studies start at high certainty in the GRADE framework. Certainty may be downgraded according to study limitations, including risk of bias, inconsistency, indirectness, imprecision, and publication bias. In the present table, the suggested certainty rating was determined primarily based on study design and the previously assessed RoB 2.0 results. Because several included studies used crossover designs, their certainty was interpreted cautiously. These ratings should be considered preliminary, as formal GRADE assessment is ideally conducted at the outcome level across studies rather than at the individual study level.

Supplementary Table 8. Preliminary GRADE assessment for non-randomized studies

| **Study** | **Initial certainty** | **Main reason for downgrading** | **Suggested GRADE certainty** |
| --- | --- | --- | --- |
| El-Tamawy (2014, Egypt) | Low | Serious risk of bias due to non-random allocation and confounding | Very low |
| de Morais (2017, Brazil) | Low | No control group and substantial risk of confounding | Very low |
| Charalambous (2018, America) | Low | Serious risk of bias due to unclear allocation and confounding | Very low |
| Harnish (2018, America) | Low | No parallel control group and limited external validity | Very low |
| King (2019, Canada) | Low | Observational design with possible selection bias and confounding | Low |
| Ryan (2019, America) | Low | No control group and limited causal inference | Very low |
| Koroleva (2020, Russia) | Low | Serious risk of bias due to non-randomized group allocation | Very low |
| Shi (2023, China) | Low | Retrospective design, selection bias, and residual confounding | Very low |

Notes: Non-randomized studies start at low certainty in the GRADE framework. Certainty may be further downgraded because of serious methodological limitations, particularly risk of bias, confounding, selection bias, indirectness, and imprecision. In the present table, the suggested certainty rating was determined primarily based on study design and the previously assessed ROBINS-I, JBI, or NOS results. These ratings should be considered preliminary, as formal GRADE assessment is ideally conducted at the outcome level across studies rather than at the individual study level.

Supplementary Table 9. Summary of predicted effects for acute total exercise dose across low-dose levels

| **Agent** | **Dose** | **Mean** | **SD** | 0.025 | 0.25 | 0.5 | 0.75 | 0.975 |
| --- | --- | --- | --- | --- | --- | --- | --- | --- |
| Placebo | 0 | 0.1674508 | 0.1030569 | 0.02225128 | 0.0890539 | 0.1484704 | 0.2291473 | 0.4034026 |
| Overall exercise | 0 | 0.1674508 | 0.1030569 | 0.02225128 | 0.0890539 | 0.1484704 | 0.2291473 | 0.4034026 |
| Overall exercise | 28 | 0.2235537 | 0.2461807 | -0.27035551 | 0.09745532 | 0.2164821 | 0.3427164 | 0.7429367 |
| Overall exercise | 56 | 0.272595 | 0.4032508 | -0.57483592 | 0.08849708 | 0.2682693 | 0.448454 | 1.1435723 |
| Overall exercise | 83 | 0.313197 | 0.5059279 | -0.72550113 | 0.0913887 | 0.3054552 | 0.5255093 | 1.435992 |
| Overall exercise | 110 | 0.3472329 | 0.5586304 | -0.76788067 | 0.10006858 | 0.3367627 | 0.5772523 | 1.5985091 |
| Overall exercise | 140 | 0.3773494 | 0.5628014 | -0.77021746 | 0.12287105 | 0.3653722 | 0.6073096 | 1.6237588 |
| Overall exercise | 170 | 0.3993596 | 0.5225697 | -0.66630383 | 0.16460225 | 0.3881686 | 0.61306 | 1.5241627 |
| Overall exercise | 190 | 0.4095294 | 0.4864663 | -0.58364591 | 0.18630276 | 0.3985307 | 0.6105734 | 1.4511847 |
| Overall exercise | 220 | 0.4180289 | 0.4698243 | -0.52441785 | 0.19050279 | 0.4077536 | 0.6233633 | 1.4554105 |
| Overall exercise | 250 | 0.418422 | 0.5791591 | -0.74443684 | 0.13819996 | 0.4096305 | 0.693503 | 1.682801 |

Supplementary Table 10. Summary of predicted effects across dose levels for long-term total exercise dose

| **Agent** | **Dose** | **Mean** | **SD** | **2.50%** | **25%** | **50%** | **75%** | **97.50%** |
| --- | --- | --- | --- | --- | --- | --- | --- | --- |
| Placebo | 0 | 0.1661777 | 0.1010907 | 0.02232219 | 0.08961322 | 0.1474808 | 0.2239935 | 0.3981513 |
| Overall exercise | 0 | 0.1661777 | 0.1010907 | 0.02232219 | 0.08961322 | 0.1474808 | 0.2239935 | 0.3981513 |
| Overall exercise | 330 | 0.1946377 | 0.1126534 | 0.009003196 | 0.11735966 | 0.1813483 | 0.261311 | 0.4448767 |
| Overall exercise | 670 | 0.2268275 | 0.1330146 | -0.013058015 | 0.13797146 | 0.2175055 | 0.3109762 | 0.5136262 |
| Overall exercise | 1000 | 0.2608536 | 0.1472588 | -0.016830132 | 0.16252432 | 0.2526604 | 0.3537173 | 0.5689963 |
| Overall exercise | 1300 | 0.2941653 | 0.1531007 | 0.007482076 | 0.19163306 | 0.2866672 | 0.3935083 | 0.6145518 |
| Overall exercise | 1700 | 0.3421053 | 0.1542411 | 0.056814177 | 0.23767158 | 0.335866 | 0.4423581 | 0.6644187 |
| Overall exercise | 2000 | 0.3807037 | 0.1579781 | 0.086642498 | 0.27278673 | 0.3759035 | 0.4809277 | 0.7035524 |
| Overall exercise | 2300 | 0.4215677 | 0.1745805 | 0.097163177 | 0.30092134 | 0.4159068 | 0.5332816 | 0.775921 |
| Overall exercise | 2700 | 0.4795775 | 0.2296497 | 0.043401293 | 0.33197051 | 0.4715473 | 0.6304647 | 0.9363958 |
| Overall exercise | 3000 | 0.5257281 | 0.2974615 | -0.031336807 | 0.33444373 | 0.5219221 | 0.7190218 | 1.1186577 |

Supplementary Table 11. Summary of predicted effects across different long-term exercise modalities and dose combinations

| **Agent** | **Dose** | **Mean** | **SD** | **2.50%** | **25%** | **50%** | **75%** | **97.50%** |
| --- | --- | --- | --- | --- | --- | --- | --- | --- |
| Placebo | 0 | 0.1677049 | 0.1035186 | 0.0241906 | 0.088178521 | 0.1483625 | 0.2319543 | 0.4149817 |
| Bicycle | 0 | 0.1677049 | 0.1035186 | 0.0241906 | 0.088178521 | 0.1483625 | 0.2319543 | 0.4149817 |
| Bicycle | 190 | 0.2903768 | 0.183019 | -0.061984136 | 0.169226218 | 0.2855362 | 0.411251 | 0.6602045 |
| Bicycle | 390 | 0.388581 | 0.2834781 | -0.168322059 | 0.197456781 | 0.3903904 | 0.5822261 | 0.9533299 |
| Bicycle | 580 | 0.4524972 | 0.3445351 | -0.220221089 | 0.215644706 | 0.4544173 | 0.6875413 | 1.1290712 |
| Bicycle | 780 | 0.4888533 | 0.3684966 | -0.223433764 | 0.241574289 | 0.4876113 | 0.7401968 | 1.2056757 |
| Bicycle | 970 | 0.4940137 | 0.355653 | -0.182470743 | 0.25898305 | 0.4953042 | 0.7338579 | 1.1971197 |
| Bicycle | 1200 | 0.4619623 | 0.3080744 | -0.136267841 | 0.257811635 | 0.4610549 | 0.6682757 | 1.0640279 |
| Bicycle | 1400 | 0.3999957 | 0.277266 | -0.131198386 | 0.208791344 | 0.3991711 | 0.584303 | 0.9396423 |
| Bicycle | 1600 | 0.3063122 | 0.3297334 | -0.311964064 | 0.087054428 | 0.2947239 | 0.5184596 | 0.9632583 |
| Bicycle | 1800 | 0.1809117 | 0.4941307 | -0.733534182 | -0.165474584 | 0.1568695 | 0.4958365 | 1.2181255 |
| Exercise combined with cognitive training | 0 | 0.1677049 | 0.1035186 | 0.0241906 | 0.088178521 | 0.1483625 | 0.2319543 | 0.4149817 |
| Exercise combined with cognitive training | 190 | 0.213184 | 0.1657535 | -0.09417902 | 0.100710893 | 0.2075332 | 0.3179225 | 0.5641009 |
| Exercise combined with cognitive training | 390 | 0.2506087 | 0.2503913 | -0.244103546 | 0.085682164 | 0.2439252 | 0.4144556 | 0.7535699 |
| Exercise combined with cognitive training | 580 | 0.2762367 | 0.3025703 | -0.320557606 | 0.078727699 | 0.2717548 | 0.4732038 | 0.8776605 |
| Exercise combined with cognitive training | 780 | 0.2927657 | 0.3219658 | -0.339899137 | 0.082617937 | 0.284776 | 0.4992526 | 0.9336315 |
| Exercise combined with cognitive training | 970 | 0.2985427 | 0.3067409 | -0.302520936 | 0.097222002 | 0.2945344 | 0.4969052 | 0.9177145 |
| Exercise combined with cognitive training | 1200 | 0.2925966 | 0.2500723 | -0.183126731 | 0.124024881 | 0.2879609 | 0.4570703 | 0.7966331 |
| Exercise combined with cognitive training | 1400 | 0.2759066 | 0.1883429 | -0.080163294 | 0.147282532 | 0.2727255 | 0.4002532 | 0.6541934 |
| Exercise combined with cognitive training | 1600 | 0.2485007 | 0.1917015 | -0.124324358 | 0.116164884 | 0.2493405 | 0.3776241 | 0.6269921 |
| Exercise combined with cognitive training | 1800 | 0.2103791 | 0.321663 | -0.419784512 | -0.004336016 | 0.2085965 | 0.4200133 | 0.8349878 |
| Multicomponent exercise | 0 | 0.1677049 | 0.1035186 | 0.0241906 | 0.088178521 | 0.1483625 | 0.2319543 | 0.4149817 |
| Multicomponent exercise | 330 | 0.4508686 | 0.2160724 | 0.042296669 | 0.308998253 | 0.4476129 | 0.5934997 | 0.8949817 |
| Multicomponent exercise | 670 | 0.679752 | 0.3492664 | -0.002514504 | 0.455227176 | 0.673327 | 0.9159128 | 1.37 |
| Multicomponent exercise | 1000 | 0.8408915 | 0.4384987 | -0.025389883 | 0.56259988 | 0.835486 | 1.1348559 | 1.68 |
| Multicomponent exercise | 1300 | 0.9352278 | 0.481807 | -0.025229379 | 0.628412128 | 0.9330322 | 1.2567544 | 1.87 |
| Multicomponent exercise | 1700 | 0.9837441 | 0.4842755 | 0.031261336 | 0.672314903 | 0.9879286 | 1.3102565 | 1.93 |
| Multicomponent exercise | 2000 | 0.9621822 | 0.4479012 | 0.087902009 | 0.668393522 | 0.9666433 | 1.2546828 | 1.85 |
| Multicomponent exercise | 2300 | 0.8909498 | 0.3864764 | 0.125286188 | 0.636626115 | 0.8923438 | 1.1330666 | 1.67 |
| Multicomponent exercise | 2700 | 0.7187077 | 0.3057093 | 0.142077295 | 0.51781673 | 0.7116535 | 0.9199891 | 1.35 |
| Multicomponent exercise | 3000 | 0.5315771 | 0.3217091 | -0.09435227 | 0.32435943 | 0.5377116 | 0.7399022 | 1.17 |
| Walking | 0 | 0.1677049 | 0.1035186 | 0.0241906 | 0.088178521 | 0.1483625 | 0.2319543 | 0.4149817 |
| Walking | 56 | 0.1824227 | 0.1499224 | -0.091318545 | 0.078896007 | 0.1734192 | 0.2806534 | 0.5014703 |
| Walking | 110 | 0.1946268 | 0.2135513 | -0.205504684 | 0.05150756 | 0.1880686 | 0.3325692 | 0.6220344 |
| Walking | 170 | 0.2058975 | 0.2646561 | -0.290684942 | 0.026827089 | 0.2005744 | 0.381126 | 0.7316872 |
| Walking | 220 | 0.2134489 | 0.286963 | -0.328953288 | 0.018167627 | 0.209895 | 0.4044451 | 0.7779946 |
| Walking | 280 | 0.2203016 | 0.2903727 | -0.33060583 | 0.023557082 | 0.2176839 | 0.4173838 | 0.7905457 |
| Walking | 330 | 0.2241714 | 0.2778288 | -0.327021241 | 0.035984503 | 0.2201331 | 0.4137288 | 0.7741769 |
| Walking | 390 | 0.2266061 | 0.2570513 | -0.294377914 | 0.059822409 | 0.2233178 | 0.4002201 | 0.7279371 |
| Walking | 440 | 0.2267941 | 0.2554826 | -0.27506319 | 0.055270564 | 0.2280612 | 0.4049148 | 0.7241689 |
| Walking | 500 | 0.2248108 | 0.3057803 | -0.374300353 | 0.017820978 | 0.2298037 | 0.4415245 | 0.7988054 |

**Included literature:**

(Bermudo-Gallaguet et al., 2025; Boyne et al., 2020; Charalambous et al., 2018; de las Heras et al., 2025; Du et al., 2021; El-Tamawy et al., 2014; García-Salazar et al., 2025; Gorna et al., 2025; Harnish et al., 2018; Hsu et al., 2021; Hu et al., 2022; Huang et al., 2022; King et al., 2019; Koroleva et al., 2020; Mackay et al., 2021; Mackay et al., 2026; Maguire et al., 2023; Morais et al., 2018; Ploughman et al., 2019; Rahayu et al., 2020; Ryan et al., 2019; Shi et al., 2023; Valkenborghs et al., 2019)

Bermudo-Gallaguet, A., Ariza, M., Agudelo, D., Camins-Vila, N., Boldo, M., Peters, S., Sawicka, A. K., Dacosta-Aguayo, R., Soriano-Raya, J. J., Via, M., Clemente, I. C., Garcia-Molina, A., Mata, M. J. D., Torán-Monserrat, P., Erickson, K. I., & Mataró, M. (2025, Apr 9). Effects of Mindfulness and Exercise on Growth Factors, Inflammation, and Stress Markers in Chronic Stroke: The MindFit Project Randomized Clinical Trial. *Journal of Clinical Medicine, 14*(8), Article 2580. <https://doi.org/10.3390/jcm14082580>

Boyne, P., Meyrose, C., Westover, J., Whitesel, D., Hatter, K., Reisman, D. S., Carl, D., Khoury, J. C., Gerson, M., Kissela, B., & Dunning, K. (2020, Mar). Effects of Exercise Intensity on Acute Circulating Molecular Responses Poststroke. *Neurorehabil Neural Repair, 34*(3), 222-234. <https://doi.org/10.1177/1545968319899915>

Charalambous, C. C., Alcantara, C. C., French, M. A., Li, X., Matt, K. S., Kim, H. E., Morton, S. M., & Reisman, D. S. (2018, May 15). A single exercise bout and locomotor learning after stroke: physiological, behavioural, and computational outcomes. *J Physiol, 596*(10), 1999-2016. <https://doi.org/10.1113/jp275881>

de las Heras, B., Rodrigues, L., Cristini, J., Yu, E. R., Gan-Or, Z., Arbour, N., Thiel, A., Tang, A. D., Fung, J., Eng, J. J., & Roig, M. (2025, Aug). Investigating the Acute and Chronic Effects of Cardiovascular Exercise on Brain-Derived Neurotrophic Factor in Early Subacute Stroke. *Neurorehabilitation and Neural Repair, 39*(8), 653-665. <https://doi.org/10.1177/15459683251342150>

Du, X., Chen, W., Zhan, N., Bian, X., & Wenbing, Y. (2021, 08/28). The effects of low-intensity resistance training with or without blood flow restriction on serum BDNF, VEGF and perception in patients with post-stroke depression. *Neuro endocrinology letters, 42*, 229-235.

El-Tamawy, M. S., Abd-Allah, F., Ahmed, S. M., Darwish, M. H., & Khalifa, H. A. (2014). Aerobic exercises enhance cognitive functions and brain derived neurotrophic factor in ischemic stroke patients. *NeuroRehabilitation, 34*(1), 209-213. <https://doi.org/10.3233/nre-131020>

García-Salazar, L. F., Pereira, N. D., Silva, E. S. M., Ribeiro, J. A. M., Ocamoto, G. N., Zambetta, R. M., de Oliveira, S. G., Catai, A. M., Borstad, A., & Russo, T. L. (2025, Jun 3). Could aerobic exercise applied before constraint-induced movement therapy change circulating molecular biomarkers in chronic post-stroke? *Physiotherapy Theory and Practice, 41*(6), 1191-1202. <https://doi.org/10.1080/09593985.2024.2411311>

Gorna, S., Podgórski, T., Kleka, P., & Domaszewska, K. (2025, Mar 20). Effects of Different Intensities of Endurance Training on Neurotrophin Levels and Functional and Cognitive Outcomes in Post-Ischaemic Stroke Adults: A Randomised Clinical Trial. *International Journal of Molecular Sciences, 26*(6), Article 2810. <https://doi.org/10.3390/ijms26062810>

Harnish, S. M., Rodriguez, A. D., Blackett, D. S., Gregory, C., Seeds, L., Boatright, J. H., & Crosson, B. (2018, Jan). Aerobic Exercise as an Adjuvant to Aphasia Therapy: Theory, Preliminary Findings, and Future Directions. *Clin Ther, 40*(1), 35-48.e36. <https://doi.org/10.1016/j.clinthera.2017.12.002>

Hsu, C. C., Fu, T. C., Huang, S. C., Chen, C. P., & Wang, J. S. (2021, Jul). Increased serum brain-derived neurotrophic factor with high-intensity interval training in stroke patients: A randomized controlled trial. *Ann Phys Rehabil Med, 64*(4), 101385. <https://doi.org/10.1016/j.rehab.2020.03.010>

Hu, Y. J., Tian, J. J., Wen, X. L., Lu, C., & Tian, N. N. (2022, May-Jun). Clinical Effects of MOTOmed Intelligent Exercise Training Combined with Intensive Walking Training on the Rehabilitation of Walking, Nerve and Lower Limb Functions among Patients with Hemiplegia after Stroke. *Pakistan Journal of Medical Sciences, 38*(5), 1222-1227. <https://doi.org/10.12669/pjms.38.5.5259>

Huang, C. Y., Chiang, W. C., Yeh, Y. C., Fan, S. C., Yang, W. H., Kuo, H. C., & Li, P. C. (2022, Jan 11). Effects of virtual reality-based motor control training on inflammation, oxidative stress, neuroplasticity and upper limb motor function in patients with chronic stroke: a randomized controlled trial. *Bmc Neurology, 22*(1), Article 21. <https://doi.org/10.1186/s12883-021-02547-4>

King, M., Kelly, L. P., Wallack, E. M., Hasan, S. M. M., Kirkland, M. C., Curtis, M. E., Chatterjee, T., McCarthy, J., & Ploughman, M. (2019, Apr). Serum levels of insulin-like growth factor-1 and brain-derived neurotrophic factor as potential recovery biomarkers in stroke. *Neurol Res, 41*(4), 354-363. <https://doi.org/10.1080/01616412.2018.1564451>

Koroleva, E. S., Tolmachev, I. V., Alifirova, V. M., Boiko, A. S., Levchuk, L. A., Loonen, A. J. M., & Ivanova, S. A. (2020, Sep 9). Serum BDNF's Role as a Biomarker for Motor Training in the Context of AR-Based Rehabilitation after Ischemic Stroke. *Brain Sci, 10*(9). <https://doi.org/10.3390/brainsci10090623>

Mackay, C. P., Brauer, S. G., Kuys, S. S., Schaumberg, M. A., & Leow, L. A. (2021). The acute effects of aerobic exercise on sensorimotor adaptation in chronic stroke. *Restor Neurol Neurosci, 39*(5), 367-377. <https://doi.org/10.3233/rnn-211175>

Mackay, C. P., Kuys, S., Schaumberg, M., Leow, L., & Brauer, S. (2026). Aerobic exercise increases brain-derived neurotrophic factor (BDNF) in sub-acute stroke: a randomized controlled trial. *Topics in Stroke Rehabilitation*. <https://doi.org/10.1080/10749357.2026.2628540>

Maguire, C., Betschart, M., Pohl, J., Primani, F., Taeymans, J., & Hund-Georgiadis, M. (2023, 2023). Effects of moderate-intensity aerobic exercise on serum BDNF and motor learning in the upper-limb in patients after chronic-stroke: A randomized, controlled feasibility study with embedded health economic evaluation. *NeuroRehabilitation, 52*(3), 485-506. <https://doi.org/10.3233/nre-220239>

Morais, V. A. C., Tourino, M., Almeida, A. C. S., Albuquerque, T. B. D., Linhares, R. C., Christo, P. P., Martinelli, P. M., & Scalzo, P. L. (2018, Jan). A single session of moderate intensity walking increases brain-derived neurotrophic factor (BDNF) in the chronic post-stroke patients. *Top Stroke Rehabil, 25*(1), 1-5. <https://doi.org/10.1080/10749357.2017.1373500>

Ploughman, M., Eskes, G. A., Kelly, L. P., Kirkland, M. C., Devasahayam, A. J., Wallack, E. M., Abraha, B., Hasan, S. M. M., Downer, M. B., Keeler, L., Wilson, G., Skene, E., Sharma, I., Chaves, A. R., Curtis, M. E., Bedford, E., Robertson, G. S., Moore, C. S., McCarthy, J., & Mackay-Lyons, M. (2019, Mar). Synergistic Benefits of Combined Aerobic and Cognitive Training on Fluid Intelligence and the Role of IGF-1 in Chronic Stroke. *Neurorehabil Neural Repair, 33*(3), 199-212. <https://doi.org/10.1177/1545968319832605>

Rahayu, U. B., Wibowo, S., Setyopranoto, I., & Hibatullah Romli, M. (2020). Effectiveness of physiotherapy interventions in brain plasticity, balance and functional ability in stroke survivors: A randomized controlled trial. *NeuroRehabilitation, 47*(4), 463-470. <https://doi.org/10.3233/nre-203210>

Ryan, A. S., Xu, H., Ivey, F. M., Macko, R. F., & Hafer-Macko, C. E. (2019, Jun). Brain-derived neurotrophic factor, epigenetics in stroke skeletal muscle, and exercise training. *Neurol Genet, 5*(3), e331. <https://doi.org/10.1212/nxg.0000000000000331>

Shi, J., Ma, S. J., Hu, J., Hu, Z. K., Xia, J. Y., & Xu, H. Y. (2023, 2023). The effects of computer-aided cognitive rehabilitation combined with virtual reality technology on event-related potential P300 and cognitive function of patients with cognitive impairment after stroke. *European Review for Medical and Pharmacological Sciences, 27*(19), 8993-9000. <Go to ISI>://WOS:001096798300003

Valkenborghs, S. R., van Vliet, P., Nilsson, M., Zalewska, K., Visser, M. M., Erickson, K. I., & Callister, R. (2019, Jul). Aerobic exercise and consecutive task-specific training (AExaCTT) for upper limb recovery after stroke: A randomized controlled pilot study. *Physiother Res Int, 24*(3), e1775. <https://doi.org/10.1002/pri.1775>
